# Engineering xylose metabolism in triacylglycerol-producing *Rhodococcus opacus* for lignocellulosic fuel production

**DOI:** 10.1186/1754-6834-6-134

**Published:** 2013-09-16

**Authors:** Kazuhiko Kurosawa, Sandra J Wewetzer, Anthony J Sinskey

**Affiliations:** 1 Department of Biology, Massachusetts Institute of Technology, 77 Massachusetts Avenue, Cambridge, MA 02139, USA; 2 Engineering Systems Division, Massachusetts Institute of Technology, 77 Massachusetts Avenue, Cambridge, MA 02139, USA; 3 Health Sciences Technology Division, Massachusetts Institute of Technology, 77 Massachusetts Avenue, Cambridge, MA 02139, USA; 4 Present address: Department of Biochemical Engineering, RWTH Aachen University, Worringerweg 1, 52074, Aachen, Germany

**Keywords:** *Rhodococcus opacus*, *Streptomyces padanus*, Triacylglycerol, Lipid-based biofuel, Lignocellulosic fuel, Lignocellulosic biomass, Xylose, High-cell-density fermentation, *xylA*, *xylB*

## Abstract

**Background:**

There has been a great deal of interest in fuel productions from lignocellulosic biomass to minimize the conflict between food and fuel use. The bioconversion of xylose, which is the second most abundant sugar present after glucose in lignocellulosic biomass, is important for the development of cost effective bioprocesses to fuels. *Rhodococcus opacus* PD630, an oleaginous bacterium, accumulates large amounts of triacylglycerols (TAGs), which can be processed into advanced liquid fuels. However, *R. opacus* PD630 does not metabolize xylose.

**Results:**

We generated DNA libraries from a *Streptomyces* bacterium capable of utilizing xylose and introduced them into *R. opacus* PD630. Xsp8, one of the engineered strains, was capable of growing on up to 180 g L^-1^ of xylose. Xsp8 grown in batch-cultures derived from unbleached kraft hardwood pulp hydrolysate containing 70 g L^-1^ total sugars was able to completely and simultaneously utilize xylose and glucose present in the lignocellulosic feedstock, and yielded 11.0 g L^-1^ of TAGs as fatty acids, corresponding to 45.8% of the cell dry weight. The yield of total fatty acids per gram of sugars consumed was 0.178 g, which consisted primarily of palmitic acid and oleic acid. The engineered strain Xsp8 was introduced with two heterologous genes from *Streptomyces*: *xylA*, encoding xylose isomerase, and *xylB*, encoding xylulokinase. We further demonstrated that in addition to the introduction and the concomitant expression of heterologous *xylA* and *xylB* genes, there is another molecular target in the *R. opacus* genome which fully enables the functionality of *xylA* and *xylB* genes to generate the robust xylose-fermenting strain capable of efficiently producing TAGs at high xylose concentrations.

**Conclusion:**

We successfully engineered a *R. opacus* strain that is capable of completely utilizing high concentrations of xylose or mixed xylose/glucose simultaneously, and substantiated its suitability for TAG production. This study demonstrates that the engineered strain possesses a key trait of converters for lipid-based fuels production from lignocellulosic biomass.

## Background

The rises in petroleum prices along with depleting petroleum reserves have kindled a great interest in the production of bio-based alternative fuels [1-3]. Extensive discussion towards the development of clean and sustainable energy sources [4], especially from lignocellulosic biomass, has occurred [5,6]. Advanced liquid (lipid-based) fuels such as gasoline, kerosene, diesel and jet fuels are of greatest interest and are expected to play a crucial role in the future global energy infrastructure [7,8]. Triacylglycerols (TAGs) are esters in which three molecules of fatty acids are linked to glycerol, and are exploited as precursors for the production of the lipid-based biofuels [9-11]. Recently, an F-22 Raptor successfully flew on a 50/50 blend of the renewable (hydroprocessed esters and fatty acids) jet fuel and conventional petroleum-based JP-8 [6]. Many sources of TAGs have been successfully converted to aviation biofuel and the consensus is that generally any TAGs (with the exception of those with very short chain fatty acids) are acceptable as precursors for fuel production [12]. Therefore, these energy-rich TAG molecules have attracted much attention for developing sustainable and high-quality fuels. Currently the main sources for TAGs are vegetable oils, animal fats or waste cooking oils [13,14]. Biofuels produced from crop seeds have come under major scrutiny due to the food vs. fuel competition issue [15]. In addition, although algae have been considered as an attractive biocatalyst for lipid-based fuels owing to their ability to produce substantial amounts of TAGs [16,17], it is currently estimated that large-scale production of biofuels from algae is unsustainable using existing technologies [18,19]. Presently, the limited supply of bioresources for obtaining TAGs is a major obstacle for the production of lipid-based biofuels, in spite of the advantageous impacts that commercialization of TAG-based biofuels could provide.

*R. opacus* PD630, an oleaginous hydrocarbon-degrading bacterium, is able to utilize long-chain-length alkanes, acetate, phenylacetic acid, phenyldecane, propionate and gluconate and produces remarkably high amounts of TAGs as a storage material [20-22]. Although TAG production of *R. opacus* PD630 on glucose as a carbon source had not been shown until recently, we have demonstrated that the strain has the rare ability to accumulate large amounts of TAGs in batch-cultivations containing high concentrations of glucose under defined conditions [23,24]. While lignocellulosic carbohydrate fractions are composed primarily of glucose, xylose represents a non-negligible portion of the sugar fraction [25]. *R. opacus* PD630 is not able to utilize xylose naturally. The effective fermentation of xylose is necessary to develop economically feasible processes for fuel productions from lignocellulosic biomass [26,27]. Expressing *xylA*, encoding xylose isomerase, and *xylB*, encoding xylulokinase, has proven to be a successful strategy to enable growth on xylose for improvement of xylose metabolism in bacteria [28-30]. Indeed, it has recently been reported that the two well-characterized genes *xylA* and *xylB* from *Streptomyces lividans* TK23 were expressed in *R. jostii* RHA1 and *R. opacus* PD630 to provide them with a xylose utilization capability and the role of the two genes in the *Rhodococcus* transformants was investigated [31].

Herein, we engineered a xylose-fermenting *R. opacus* strain capable of high-cell-density cultivation at high xylose concentrations and substantiated its usefulness for TAG production using genuine lignocellulosic hydrolysate. The present study also provides evidence for the existence of the underlying cryptic molecular target responsible for improving xylose metabolism, in addition to the expression of heterologous *xylA* and *xylB* genes, to engineer a robust xylose-fermenting *Rhodococcus* strain.

## Results

### Metabolic engineering of *R. opacus* PD630 to produce TAGs on xylose

In order to engineer a xylose utilizing strain of *R. opacus* PD630, we sought to transfer the genes encoding enzymes essential for xylose metabolism. *Streptomyces padanus* MITKK-103, an actinomycete that is closely related to *Rhodococcus*, efficiently utilizes xylose as a sole carbon source [32]. *S. padanus* genomic DNA was partially digested with the restriction enzyme Sau3AI and the DNA fragments were ligated into the BamHI site of the pAL358 plasmid bearing a gentamicin resistance cassette and an origin of replication that allows for propagation in *R. opacus*. *R. opacus* PD630 was transformed by electroporation using the resulting plasmid libraries from *S. padanus*. A total of 42 transformants were isolated and grown in a defined medium containing xylose as a sole carbon source. Although the cell growth and morphology of those strains on the plates were robust and similar, curiously, the capabilities of utilizing xylose and producing TAGs when grown in the flasks were different from one another. Eight strains were capable of growing in the flasks, reaching an OD_660_ greater than 10, and only 4 of those, termed Xsp1, 8, 10 and 12, accumulated over 30% of the cell dry weight (CDW) as TAGs (Additional file 1: Table S1).

### Growth of *R. opacus* Xsp8 with high xylose concentrations

**Figure 1:**
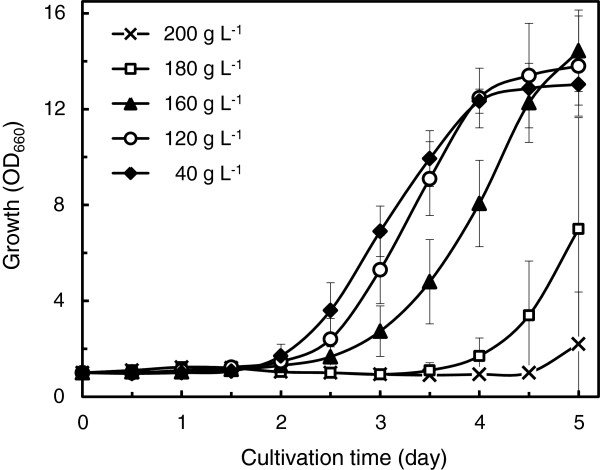
**Growth kinetics of *R. opacus *Xsp8 on high xylose concentrations in flask cultures**. Xylose concentrations of modified defined media with 1.4 g L^-1^ (NH_4_)_2_SO_4_ and 10 mg L^-1^ gentamicin were 40, 120, 160, 180 and 200 g L^-1^. Initial inoculum densities were adjusted photometrically to obtain an OD_660_ of 1.0. Values and error bars represent the mean and s.d. of triplicate experiments.

High-cell-density cultivation is a prerequisite to maximize volumetric productivity of microbial oil fermentation, and the media used should be typically composed of highly concentrated sugars [33-35]. We examined the growth kinetics of strain Xsp8, the transformant that revealed the highest level of fatty acid production, on defined medium with initial xylose concentrations of 40, 120, 160, 180 and 200 g L^-1^ in flask cultures (Figure 1). Xsp8 grew on media containing up to 180 g L^-1^ of xylose, reaching stationary phase after 4 days on 40 and 120 g L^-1^ and 5 days on 160 g L^-1^, respectively.

### TAG production of *R. opacus* Xsp8 on xylose and/or glucose

**Figure 2:**
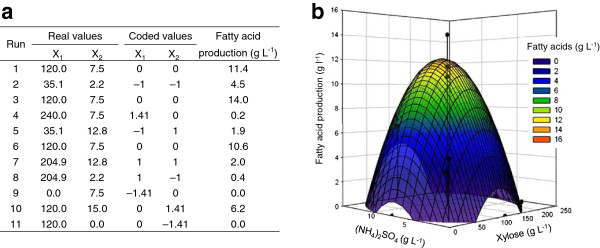
**Optimization of TAG production from xylose by *R. opacus *Xsp8 in batch-culture fermentations. (a)** Central composite experimental design matrix defining xylose and (NH_4_)_2_SO_4_ concentrations. X_1_, xylose concentration (g L^-1^); X_2_, (NH_4_)_2_SO_4_ concentration (g L^-1^). The strain was inoculated in the modified defined medium supplemented with gentamicin in Sixfors-bioreactors. The data for TAG production as fatty acids represent the maximum values during 10 days of cultivation. **(b)** Response surface plot of the effect of xylose and (NH_4_)_2_SO_4_ concentrations on fatty acid production. Curves and points represent predicted values and experimental data, respectively.

We have previously demonstrated that *R. opacus* PD630 accumulates large amounts of TAGs at a critical carbon to nitrogen ratio (C/N) [23]. The operational C/N (w/w) ratio of xylose and (NH_4_)_2_SO_4_ in the defined medium for maximum production of TAGs (as fatty acids) by Xsp8 strain was optimized using response surface methodology [36]. The experimental design model determined nine combinations of xylose and (NH_4_)_2_SO_4_ concentrations with a central point (120 g L^-1^ xylose and 7.5 g L^-1^ (NH_4_)_2_SO_4_) to be run in triplicate for a total of 11 bioreactors in batch fermentations (Figure 2a). The analysis by the StatGraphics software showed a high regression coefficient (R^2^=0.9202) and the equation of the fitted model is:

$$\begin{array}{l} Y=-6.815+0.1701X_{1}+2.199X_{2}–0.0008047X_{1}{}^{2}\\ \;+0.002333X_{1}X_{2}–0.1531X_{2}{}^{2} \end{array}$$

Where Y is the TAG production as fatty acids (g L^-1^); and X_1_ and X_2_ are the uncoded values of xylose and (NH_4_)_2_SO_4_ concentrations (g L^-1^), respectively. The values of Y based on the range of X_1_ and X_2_ were calculated for all fermentations and illustrated in a response surface plot (Figure 2b). The design predicted a maximum production of fatty acids of 12.1 g L^-1^ by Xsp8 in the defined medium with a C/N ratio of 14.5 containing 117 g L^-1^ xylose and 8.08 g L^-1^ (NH_4_)_2_SO_4_. The operational C/N ratio for maximum production of fatty acids of Xsp8 on xylose was slightly lower than that of PD630 (C/N of 17.8) [23], the parental strain of Xsp8, on glucose.

**Figure 3:**
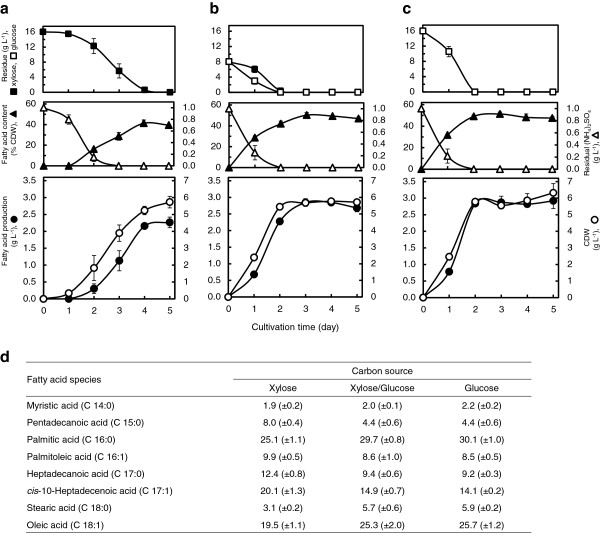
**TAG production from xylose and/or glucose by *R. opacus *Xsp8. (a-c)** Time course kinetics of TAG production as fatty acids. The strain was inoculated in modified defined medium containing 16 g L^-1^ xylose **(a)**, a mixture of 8 g L^-1^ xylose and 8 g L^-1^ glucose **(b)**, or 16 g L^-1^ glucose **(c)** with 1 g L^-1^ (NH_4_)_2_SO_4_ and gentamicin in flasks. Values and error bars represent the mean and s.d. of triplicate experiments. **(d)** Fatty acids composition profile as % of total fatty acids (g/g) of *R. opacus* growing in the defined medium containing xylose **(a)**, xylose and glucose mix **(b)**, or glucose **(c)** for 4 days. Data are results of triplicate experiments, ±s.d.

Fermentation media derived from lignocellulosic biomass are generally composed of mixtures of hexoses and pentoses, mostly glucose and xylose [25]. The successful performance of TAG fermentation depends on the ability of the producer to cope with glucose/xylose co-fermentation [37]. To elucidate the sugar-assimilation profile by Xsp8, we carried out flask cultivations in defined media containing 16 g L^-1^ xylose, a mixture of 8 g L^-1^ xylose and 8 g L^-1^ glucose, and 16 g L^-1^ glucose. The kinetics of fatty acid production, CDW, fatty acid content (as percent of CDW), and residual sugars and (NH_4_)_2_SO_4_ present in the culture supernatants were determined (Figure 3a-c). When Xsp8 was cultivated on xylose alone (Figure 3a), the growth started after 1 day of cultivation and the fatty acid accumulation increased after (NH_4_)_2_SO_4_ was depleted, and maximum fatty acid production of 2.27 (±0.16) g L^-1^ representing 39.5 (±0.62) % of CDW occurred after 5 days of cultivation, at which point the residual xylose was completely consumed. Growing on a xylose/glucose mixture (Figure 3b) and glucose alone (Figure 3c), cell growth increased rapidly after 1 day of cultivation and fatty acid production in the stationary phase 3 days post-inoculation was 2.83 (±0.13) g L^-1^ and 2.87 (±0.19) g L^-1^, respectively, corresponding to 50.0 (±2.6) % CDW and 51.6 (±2.4) % CDW, respectively. Growth kinetics of Xsp8 grown on a xylose/glucose mix showed that the culture had a shorter lag phase in comparison with that grown on xylose alone, and was similar to that grown on glucose alone. The concentrations of mixed sugars in the medium simultaneously decreased over time without diauxic behaviour, and the consumption of xylose and glucose was complete after 2 days of cultivation. During 5 days of cultivation, the maximum yield of total fatty acids per gram of sugar consumed was 0.142 (±0.010) g on xylose alone, 0.177 (±0.007) g on a xylose/glucose mix and 0.179 (±0.012) g on glucose alone, respectively. When mixed xylose-glucose substrates in the medium were used, both the maximum lipid production and lipid yield per sugar consumed were slightly lower than those attained with glucose alone, but higher than those with xylose alone. The identity of the lipids and the fatty acid composition profile of Xsp8 grown under these conditions were quite similar to one another (Figure 3d).

### Fermentation of *R. opacus* Xsp8 on unbleached kraft hardwood pulp hydrolysate

**Figure 4:**
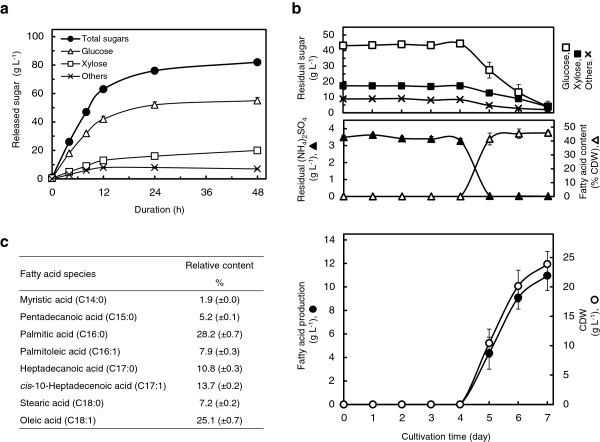
**TAG production from unbleached kraft hardwood pulp hydrolysate by *R. opacus *Xsp8 in batch-culture fermentations. (a)** Saccharification of unbleached kraft hardwood pulp using commercially available enzymes. Eighty grams of dried pulp were suspended in 1 liter deionized water and adjusted to a pH 5.0 with 0.1N HCl. Four enzymes, 2 g of Pentopan and 10 ml each of Viscozyme, Celluclast and Novozyme 188, were added into the suspension. After 4, 8 and 12 h of duration, additional 80 g of the pulp, 2 g of Pentopan and 10 ml each of the three enzymes were mixed into the suspension three times. Values and error bars represent the mean and s.d. of triplicate experiments. **(b)** Time course kinetics of TAG production as fatty acids on the pulp hydrolysate in bioreactor cultivations. Xsp8 was inoculated in the saccharified solution containing 70 g L^-1^ of total sugars composed of 47 g L^-1^ glucose, 17 g L^-1^ xylose and 6 g L^-1^ other sugars supplemented with 3.47 g L^-1^ (NH_4_)_2_SO_4_, mineral components of defined medium and gentamicin, at an initial OD_660_ of 0.5. Values and error bars represent the mean and s.d. of triplicate experiments. **(c)** Fatty acids composition profile as % of total fatty acids (g g^-1^) of TAGs from Xsp8 cells growing in the saccharified solution for 7 days. Data are results of triplicate experiments, ±s.d.

We also validated the simultaneous metabolism of xylose and glucose using genuine unbleached kraft hardwood pulp hydrolysate. Four commercial enzymes (Viscozyme, Celluclast, Novozyme 188 and Pentopan) were added to the suspension of unbleached kraft hardwood pulp adjusted to pH 5.0. After 48 h at 45°C, the released sugar was 82 (±2.3) g L^-1^ in total, composed of 55 (±2.3) g L^-1^ glucose, 20 (±1.2) g L^-1^ xylose and 7 (±0.6) g L^-1^ other monosaccharides (Figure 4a). Xsp8 was cultivated in the filtered saccharified solution containing 70 g L^-1^ initial total sugars, and supplemented with minimal nutrients (Figure 4b). The lag phase in the saccharified solution was longer than that in the refined sugar solutions (Figure 3a-c), and the cell growth increased rapidly after 4 days of cultivation. However, the depletion of nitrogen and the initiation of simultaneous utilization of xylose and glucose in the solution occurred between 4 days and 5 days, and fatty acid production of 11.0 (±1.3) g L^-1^ corresponding to 45.8 (±1.3) % of the cell dry weight was obtained after 7 days of cultivation, at which point the residual sugars were consumed. The yield of total fatty acids per gram of sugars consumed was 0.178 (±0.021) g, the accumulated fatty acids consisted primary of palmitic acid and oleic acid (Figure 4c) and the fatty acid composition profile under these conditions was very similar to those from refined sugar fermentations (Figure 3d). These results show that Xsp8 is capable of completely metabolizing both sugars simultaneously at concentrations greater than 70 g L^-1^ of a xylose/glucose mixture producing significant amounts of TAGs even when grown on lignocellulosic hydrolysates.

### Underlying molecular targets responsible for improvement of xylose metabolism in *R. opacus* PD630

To know whether the utilization of some sole carbon sources for growth is compared to the wild-type PD630, we assessed the differences of those strains in catabolism of 190 different substrates with the Biolog Phenotype MicroArrays [38]. The utilization of the individual sole carbon source was expressed as the corrected average values of color development for each substrate after 5 days of incubation (Additional file 2: Figure S1). Xsp8 significantly differed from PD630 in the catabolism of only one of the tested substrates. Xylose was not utilized by PD630, but Xsp8 did catabolize xylose. The carbon source utilization profiles of these strains were essentially unchanged, although it was observed that Xsp8 was slightly impaired in its ability to utilize D-trehalose, L-valine, L-leucine and α-ketovaleric acid, relative to the parental strain.

**Figure 5:**
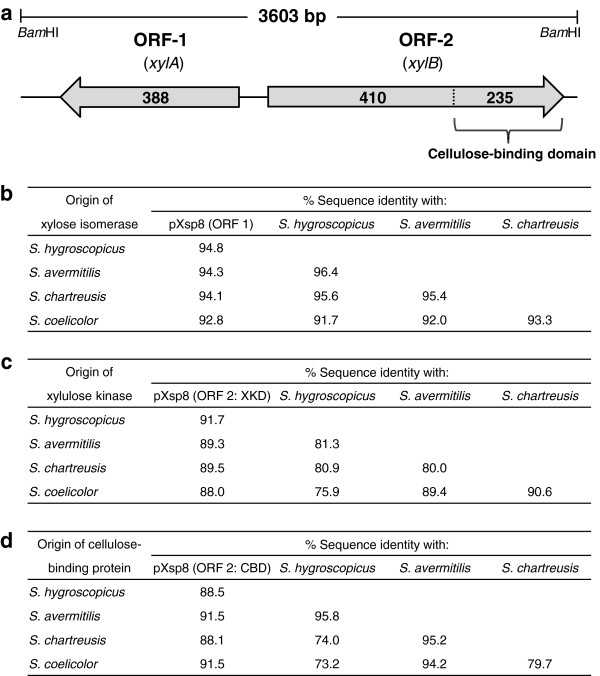
**Identification of the genes derived from *S. padanus *involved in improving xylose metabolism in *R. opacus *PD630. (a)** Physical map of the 3603-bp insert on pXsp8. The numbers and arrows in the ORF boxes indicate those of the amino acid residue and the locations of ORFs, respectively. **(b-d)** Pairwise identities of amino acid sequences using the CLUSTALW program. **(b)** The ORF 1 (388 aa) and xylose isomerases of *S. hygroscopicus* accession no. AEY87893 (388 aa), *S. avermitilis* accession no. NP_828358 (388 aa), *S. chartreusis* accession no. ZP_09957234 (388 aa) and *S. coelicolor* accession no. NP_625460 (387 aa). **(c)** Xylulose kinase domain (XKD, 410 aa) of the ORF 2 and xylulose kinases of *S. hygroscopicus* accession no. AEY87894 (481 aa), *S. avermitilis* accession no. NP_828357 (481 aa), *S. chartreusis* accession no. ZP_09957235 (481 aa) and *S. coelicolor* accession no. NP_733525 (481 aa). **(d)** Cellulose-binding domain (CBD, 235 aa) of the ORF 2 and cellulose-binding proteins of *S. hygroscopicus* accession no. AEY91743 (311 aa), *S. avermitilis* accession no. NP_824035 (312 aa), *S. chartreusis* accession no. ZP_09956458 (311 aa) and *S. coelicolor* accession no. NP_629535 (310 aa).

The plasmid was isolated, and sequenced, and designated pXsp8. Sequence analysis demonstrated that pXsp8 contained a 3603 bp insert within the pAL358 vector backbone, and the nucleotide sequence of the whole insert had two open reading frames (ORFs) (Figure 5a). ORF-1 consisted of 1164 bp, and the derived amino acid sequence composed of 388 amino acid residues was highly homologous with those of xylose isomerases (*xylA*) and was comparable in size to those from sequenced *Streptomyces* (Figure 5b, Additional file 2: Figure S2). ORF-2 comprised 1935 bp predicted to encode a xylulokinase (*xylB*) with 645 amino acid residues, and the size of the ORF-2 product was large in comparison with *xylB* homologs from other bacterial species. Interestingly, ORF-2 had two domains: the first 410 amino acids of the protein revealed a clear similarity to xylulokinases, and the last 235 C-terminus amino acids had a high homology with bacterial-like cellulose-binding domains (CBDs) (Figure 5c, d, Additional file 2: Figure S3). The 3′ region of the predicted *xylB* gene appeared to be absent and replaced by the 5′ region of a potential cellulose binding protein.

**Figure 6:**
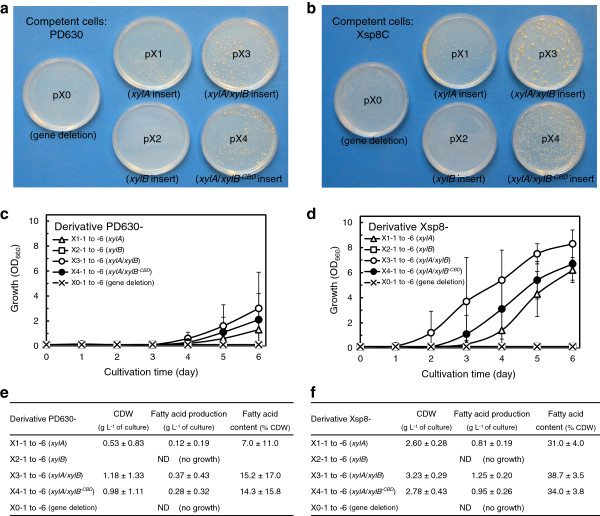
**Elucidation of the molecular targets involved in improving xylose metabolism in *R. opacus *PD630. (a,b)** Colonies arising from transformation of various plasmids (pX0 which carries no gene, pX1 which carries *xylA* alone, pX2 which carries *xylB* alone, pX3 which carries *xylA* and *xylB*, and pX4 which carried *xylA* and *xylB* without cellulose-binding domain) into *R. opacus* competent cells. Each plasmid was electroporated into the competent cells of *wt* PD630 **(a)** or pXsp8-plasmid cured Xsp8C **(b)**. The pulsed cells were plated onto defined medium containing 16 g L^-1^ xylose, 1 g L^-1^ (NH_4_)_2_SO_4_ and spectinomycin, and incubated for 14 days. **(c-f)** Fermentation kinetics of *R. opacus* derivatives. Each strain was inoculated in defined medium containing 16 g L^-1^ xylose, 1 g L^-1^ (NH_4_)_2_SO_4_ and spectinomycin at an initial OD_660_ of 0.1 in flask cultures. **(c,d)** Growth kinetics of PD630 derivatives **(c)** and Xsp8 derivatives **(d)**. **(e,f)** TAG production of PD630 derivatives **(e)** and Xsp8 derivatives **(f)** after 6 days of cultivation. Data represent the mean and s.d. of the six strains (n=1).

In order to identify the genes involved in improving xylose metabolism, the plasmids pX1 (harboring *xylA*), pX2 (harboring *xylB*), pX3 (harboring *xylA* and *xylB*), pX4 (harboring *xylA* and *xylB* without CBD) and pX0 (bearing no added genes) were constructed (Additional file 2: Figure S4) and electroporated into competent cells of *R. opacus* PD630 (*wt* strain) and Xsp8C (plasmid pXsp8-cured strain of Xsp8). The pulsed cells were incubated on a defined agar medium containing xylose as a sole carbon source. After 14 days of cultivation, faint colonies were observed on the plates spread with the pX0- and pX2-electroporated cells, but robust colonies were observed when pX1, pX3 and pX4 were transformed into the cells (Figure 6a, b). Intriguingly, colonies of competent cells of Xsp8C on the plates appeared to be larger than those of PD630, and many robust, large colonies appeared on the plates spread with the pX3-electroporated Xsp8C cells. Six colonies from each of the plates (Figure 6a, b) were randomly isolated and screened for growth and TAG production on xylose. Although the growth and morphology of the LB plate isolates were identical to each other (Additional file 2: Figure S5a, b), all of the derivatives carried the expected pX1, pX2, pX3, pX4 or pX0 plasmid (Additional file 2: Figure S5c, d). A total of 60 strains (30 each of PD630- and Xsp8-derivatives) were grown in a defined medium containing xylose as a sole carbon source. PD630- and Xsp8-derivatives carrying pX2 or pX0 showed no growth in the medium, but those carrying pX1, pX3 and pX4 were able to grow on xylose, and their growth and lipid production were as follows: transformants carrying pX3 outperformed transformants carrying pX4, which outperformed transformants carrying pX1 (Figure 6c-f). These results demonstrate that *xylA* alone confers the ability of *R. opacus* to use xylose, while *xylB* alone does not. We concluded that the introduction and expression of *xylA* is a prerequisite for improvement of xylose metabolism in *R. opacus* PD630, that *xylB* plays a synergistic role with *xylA* and the cellulose binding protein domain on *xylB* promotes the function of *xylB*. Curiously, the growth and lipid production of isolated transformants on xylose differed significantly depending on the competent cells used between PD630 (Figure 6c, e) and Xsp8C (Figure 6d, f) when the plasmid carrying *xylA* and/or *xylB* was transformed into *R. opacus*. These data can be interpreted to mean that there is at least one other molecular target involved in improving xylose metabolism in *R. opacus* in addition to the introduction and the concomitant expression of heterologous *xylA* and *xylB* genes.

## Discussion

The capability to efficiently utilize xylose as a fermentable substrate is a key attribute of microbial biocatalysts for optimizing lignocellulose-derived biofuel productions [4,5]. Despite recent extensive efforts to develop efficient industrial biotechnological schemes for deriving biofuels from lignocelluloses, the bioconversion of xylose remains a challenge [26,27]. Several microorganisms have been shown to grow well on xylose, but relatively few wild-type strains can utilize xylose [39]. *R. opacus* PD630 is unable to catabolize xylose, as expected, although it accumulates large amounts of TAGs on glucose concentrations of more than 200 g L^-1^, indicating that it holds great potential as a future source of industrial biofuels derived from renewable biomass resources [23]. Members of the genus *Rhodococcus* are actinomycetes and relatives of *Streptomyces*. We had previously disclosed that the genes from *Streptomyces* are functionally expressed in *Rhodococcus* following horizontal gene transfer from *Streptomyces* to *Rhodococcus* through competitive co-culture [40]. In this study, we manipulated a xylose metabolic pathway in *R. opacus* PD630 employing genes from *S. padanus* MITKK-103, which utilizes xylose favourably as a sole carbon source. Consequently, we constructed a xylose-fermenting *R. opacus* strain Xsp8, capable of high-cell-density fermentation and TAG production under high concentrations of xylose. Xsp8 grown in batch-cultures on 120 g L^-1^ xylose yielded 12.0 (±1.8) g L^-1^ of TAGs (Figure 2). A wild-type or an engineered *Rhodococcus* strain capable of metabolizing xylose at greater than 100 g L^-1^ has not yet been reported [31], to our knowledge. It is well known that most microorganisms preferentially utilize glucose in a mixture of glucose and xylose due to carbon catabolite repression or allosteric competition of the sugars in sugar transport [41,42]. Diauxic metabolism negatively impacts the process efficiency, indicating that the development of non-diauxic microbial strains is one of the main technological bottlenecks for lignocellulosic fuel production [37]. *R. opacus* Xsp8 was also able to completely utilize both sugars simultaneously in a 1:1 xylose/glucose mixture for growth and TAG production. The strain exhibited a shorter lag phase compared to that on xylose alone (Figure 3). In addition, Xsp8 was completely and simultaneously able to utilize xylose and glucose present in a lignocellulosic feedstock derived from unbleached kraft hardwood pulp hydrolysate containing 70 g L^-1^ total sugars. The TAG production was 11.0 g L^-1^ as fatty acids, corresponding to 45.8% of CDW (Figure 4). Our engineered *R. opacus* Xsp8-strain could potentially be the most promising biocatalyst in existence for lipid-based fuels production from lignocellulosic biomass.

We also tried to identify the genes involved in improving the xylose metabolism in *R. opacus* PD630. The engineered strain Xsp8 was introduced with two heterologous genes from *S. padanus*: *xylA*, encoding xylose isomerase, and *xylB*, encoding xylulokinase (Figure 5). Intriguingly, the 3′ region of the predicted *xylB* gene was absent, apparently replaced by the 5′ region of a predicted cellulose-binding protein. The introduction and expression of *xylA* alone were sufficient to enable *R. opacus* PD630 to utilize xylose. The *xylB* gene by itself was insufficient for improving the xylose metabolism, and played a synergistic role with *xylA* in efficiently metabolizing xylose. The kinetics of the cellulose binding domain on *xylB* needs to be investigated. These findings suggest that a partly functional pathway consisting of the second step that phosphorylates *D*-xylulose by xylulokinase (*xylB* gene) in xylose metabolism is present in the parental *R. opacus* strain PD630. Indeed, our analysis of the whole genome sequence of *R. opacus* PD630 (http://www.broadinstitute.org/) [43] revealed two open reading frames, the predicted product of which show homology to xylulokinases. Mostly two approaches have been addressed in metabolic engineering of xylose-fermenting strains: the expression of xylose isomerase and xylulokinase, encoding *xylA* and *xylB*, respectively, or the expression of three genes encoding xylose reductase, xylitol dehydrogenase and xylulokinase [28-30,44]. In this study, the former strategy, involving expression of the genes *xylA* and *xylB*, contributed to the improvement of xylose metabolism for *R. opacus* PD630. The findings are in accordance with the results that recently addressed engineering of a xylose metabolic pathway in *Rhodococcus* strains [31]. Meanwhile, we suggest that there is at least one additional molecular target in the *R. opacus* genome which fully enables the functionality of *xylA* and *xylB* genes to generate the xylose-fermenting strain capable of efficiently producing TAGs at high xylose concentrations (Figure 6). When the plasmid pX3 (harboring *xylA* and *xylB* derived from *S. padanus*) was electrotransformed into the competent cells of *R. opacus* PD630 (wt strain) and Xsp8C (plasmid pXsp8-cured strain of Xsp8) and the resulting strains were incubated on a defined agar medium containing xylose, colonies of Xsp8C cells on the plate were larger in size than those of PD630. Additionally, the production parameters of the isolated transformants in flask culture with xylose differed significantly depending on the competent cells used between PD630 and Xsp8C. The growth and TAG production of Xsp8C-derived transformants carrying pX3 were clearly superior to those of PD630-derived transformants carrying pX3. The most probable molecular mechanism accounting for this curious phenomena may be the activation of a xylose uptake system, the mutation and expression of genes encoding xylose transporters, already present in the wild-type strain, PD630. Several xylose transporters have been found in bacteria [45,46], including both the ABC-type transporter and xylose-proton symporter. We have not specified them at this point in *R. opacus* PD630, although several hypothetical proteins are annotated as possible xylose transporters in the genome of PD630 (http://www.broadinstitute.org/). Further studies are required to elucidate a mechanism of xylose uptake. These results provide important clues to develop a more robust xylose-fermenting strain for deriving low-cost biofuels from lignocellulosic biomass.

## Conclusions

In this study, we successfully engineered a *R. opacus* strain that is capable of completely utilizing high concentrations of xylose or mixed xylose/glucose simultaneously and substantiated its usability for TAG production. The creation of a robust xylose-fermenting strain required the concomitant expression of *xylA* encoding xylose isomerase and *xylB* encoding xylulokinase. An engineered xylose-fermenting *R. opacus* strain Xsp8 was capable of completely metabolizing xylose and glucose present in unbleached kraft hardwood pulp hydrolysate containing 70 g L^-1^ of total sugars producing significant amounts of TAGs.

## Methods

### Materials

LB broth, ISP medium No.1 (tryptone-yeast extract broth), nutrient broth, yeast extract and agar were purchased from BD Diagnostic Systems (Sparks, MD). Unbleached kraft hardwood pulp was kindly provided by Old Town Fuel & Fiber (Old Town, ME). Restriction enzymes, Phusion PCR Master Mix and molecular weight markers were purchased from New England Biolabs (Ipswich, MA). The Zero Blunt TOPO PCR Cloning Kit was purchased from Invitrogen and the Geneclean II Kit was purchased from MP Biomedicals (Solon, OH), and both were used according to the manufacturers’ guidelines. All other chemicals and commercial enzymes used were obtained from Sigma-Aldrich (St. Louis, MO) unless otherwise noted.

### Strains, plasmids, media and culture conditions

A list of strains and plasmids used are given in Additional file 1: Table S2. *R. opacus* PD630 (DSMZ 44193) was obtained from Deutsche Sammlung von Mikroorganismen und Zellkulturen GmbH. *S. padanus* MITKK-103 was isolated in our laboratory [32]. One Shot TOP 10 chemically competent *Escherichia coli*, which was used for gene library construction and DNA manupulation, was purchased from Invitrogen (Carlsbad, CA). Plasmids pAL358 and pAL307 which carry an origin of replication and a resistance cassette of gentamicin and spectinomycin, respectively, were used as cloning vectors [47,48].

The culture media used in this study include ISP medium No.1, LB broth, NBYE medium consisted of 8 g nutrient broth and 5 g yeast extract per liter of deionized water, and a phosphate-buffered defined medium [49] which contained per liter: 16 g xylose, 1.0 g (NH_4_)_2_SO_4_, 1.0 g MgSO_4_∙7H_2_O, 0.015 g CaCl_2_∙2H_2_O, 1.0 ml trace element solution, 1.0 ml stock A solution and 35.2 ml 1.0 M phosphate buffer. Xylose, MgSO_4_∙7H_2_O and CaCl_2_∙2H_2_O were dissolved in deionized water and sterilized by autoclaving and then stock A, trace elements, and (NH_4_)_2_SO_4_ were added to the cooled medium as filter sterilized stock solutions. Any modifications of the defined medium are stated in figure and table legends. Solid media were supplemented with 2% agar. In the case of plasmid-containing cultures, antibiotics were added to solid or liquid medium at the following final concentrations where appropriate: gentamicin at 10 mg L^-1^, spectinomycin at 100 mg L^-1^ or kanamycin at 50 mg L^-1^.

*S. padanus* was grown at 30°C in ISP medium No.1, and *E. coli* was grown at 37°C in LB broth. *R. opacus* strains were grown at 30°C in either LB broth or defined medium. *R*. *opacus* seed cultures for flask and fermentor cultivations were prepared in defined medium. A loopful of cells from a single colony grown on an LB agar plate for 3 days was used to inoculate 50 ml of defined medium in a 250-ml baffled flask. The culture was then incubated on a rotary shaker (200 rpm) until it reached an optical density (OD_660_) of 15, as determined by a Spectronic 20 Genesys spectrophotometer (Spectronic Instruments). Shake flask experiments were carried out using 250-ml baffled flasks containing 50 ml of defined medium incubated on a rotary shaker (200 rpm) at 30°C. Unless otherwise stated, cultures were inoculated with a seed culture to an initial OD_660_ of about 0.3 (7.5 × 10^7^ cfu mL^-1^). Fermentor experiments were performed with a Sixfors bioreactor system (Infors) as previously described [23]. Unless otherwise stated, fermentor-cultures were inoculated with the seed culture at an initial OD_660_ of 1.0.

### Experimental design and statistical analysis for medium optimization

The operational C/N ratio of the medium for maximum production of fatty acids was optimized using a response surface methodology based on the Box-Wilson Central Composition Design [36]. The design matrix of the experimental conditions was subjected to regression analysis by using the software StatGraphics (StatPoint). The design class chosen to carry out the experiments was a response surface with 11 proofs and a triplicate central point.

### Genetic manipulations

DNA manipulations were carried out using established methods [50], and used following the manufacturers’ instructions. Genomic DNAs from *S. padanus* and *R. opacus* were prepared as previously described [32,47]. PCR amplifications were performed with a Bio-Rad C1000 Thermal Cycler using Phusion High-Fidelity PCR kits*.* Plasmids were purified with Perfectprep plasmid mini kits. DNA fragments were recovered from agarose gels by using Geneclean II kits. Primers used in this study are described in Additional file 1: Table S3. DNA sequencing was carried out by using an Applied Biosystems Model 3730 DNA sequencer at the MIT Biopolymers Laboratory. The open reading frame (ORF) identification and similarity searches of nucleotide and protein sequences were performed using the BLASTN, BLASTX, BLASTP and ORF Finder programs from the NCBI website (http://blast.ncbi.nlm.nih.gov/Blast.cgi). Multiple sequence alignments were done with the program CLUSTALW (http://www.genome.jp/tools/clustalw/).

### Construction of *S. padanus* genomic DNA library and plasmids

For *S. padanus* plasmid library, genomic DNA of *S. padanus* MITKK-103 was partially digested with Sau3AI, and electrophoresed on an agarose gel (0.7%). The digested fragments ranging from 2 to 6 kbp were excised from the agarose gel, purified, ligated with BamHI-digested pAL358 vector and transformed into *E. coli* TOP10 competent cells. The cells were plated onto LB plates containing gentamicin, and approximately 30,000 colonies were scraped off the plates after 24 h of cultivation. The plasmid library was purified and used to transform into *R. opacus*.

For plasmid pX1, the DNA fragment encoding *xylA* gene was PCR-amplified from pXsp8 using a pair of primers 3603-*xylA*-U and 3603-*xylA*-D1, which incorporated BamHI and SpeI restriction sites. The PCR product was cloned into pCR-Blunt II-TOPO and proliferated in *E. coli* TOP10 competent cells. Positive clones were identified through selection on LB plates containing kanamycin and 50 mg L^-1^ of X-gal (5-bromo-4-chloro-indolyl-β-D-galactopyranoside). The plasmids were isolated and digested with BamHI and SpeI for ligation into pAL307 vector, which was similarly digested. The 1475-bp fragment and digested pAL307 were purified, and cohesive end ligations were carried out with T4 DNA ligase. The ligation mixtures were again transformed into *E. coli* TOP10 cells, and the positive clones were identified through selection on LB plates containing spectinomycin, and pX1 was isolated. For plasmid pX2, the fragment encoding *xylB* gene was amplified from pXsp8 using a pair of primers 3603-*xylB*-U1 and 3603-*xylB*-D, which incorporated BamHI and SpeI restriction sites. The product was digested with BamHI and SpeI and the resulting 2064-bp fragment was cloned into BamHI-SpeI-digested pAL307, as described above, creating pX2. For plasmid pX3, the fragment encoding *xylA* gene was amplified from pXsp8 using a pair of primers 3603-*xylA*-U and 3603-*xylA*-D2, which incorporated BamHI and PstI restriction sites. The product was digested with BamHI and PstI (fragment 1). The DNA encoding *xylB* gene was amplified from pX2 using a pair of primers 3603-*xylB*-U2 and 3603-*xylB*-D, which incorporated PstI and SpeI restriction sites. The product was digested with PstI and SpeI (fragment 2). Fragments 1 and 2 were then ligated together with T4 DNA ligase, and the ligated fragment was purified from an electrophoresed agarose gel. The 3902-bp fragment was cloned into BamHI-SpeI-digested pAL307, creating pX3. For plasmid pX4, the fragment encoding *xylA* gene was amplified from pXsp8 using a pair of primers 3603-*xylA*-U and 3603-*xylA*-D2, which incorporated BamHI and PstI restriction sites. The product was digested with BamHI and PstI (fragment 3). The DNA encoding *xylB* gene without cellulose-binding domain was amplified from pX2 using the primers 3603-*xylB*-U2 and 3603-*xylB*#-D, which incorporated PstI and SpeI restriction sites. The product was digested with PstI and SpeI (fragment 4). Fragments 3 and 4 were ligated, and the resulting 3191-bp fragment was cloned into BamHI-SpeI-digested pAL307, creating pX4. For plasmid pX0, the fragment of an untranslated region noncoding *xylA* and/or *xylB* genes was amplified from pXsp8 using a pair of primers 3603-*xylA*-U and 3603-*dele*-D, which incorporated BamHI and SpeI restriction sites. The product was digested with BamHI and SpeI, and the resulting 125-bp fragment was cloned into BamHI-*Spe*I-digested pAL307, creating pX0.

### Electroporation of *R. opacus*

*R. opacus* electrocompetent cells of PD630 and Xsp8C (plasmid-cured strain of Xsp8) were prepared as previously described [47]. Xsp8C was obtained by consecutively passaging individual colonies of Xsp8 on LB plates and testing for sensitivity to gentamicin and plasmid loss using agarose gel electrophoresis. *S. padanus* plasmid library, pX0, pX1, pX2, pX3 or pX4 were introduced into PD630 or Xsp8C by electroporation using a Bio-Rad Gene Pulser. Immediately before the electroporation, 70 μl of competent cells were mixed with the plasmid DNA (50~500 ng). The electrotransformation was performed in a 2 mm electroporation cuvette (VWR North American) and electroporated at 2.5 kV, 25 μF and 200 Ω. Pulsed cells were diluted with 300 μl of LB broth, regenerated for 3 h with gentle agitation, plated onto defined xylose plates containing gentamicin or spectinomycin and incubated to identify xylose utilizing transformants.

### Analytical methods

Cell growth was followed by measurement of the optical density at 660 nm (Thermo Scientific GENESYS 20) or cell dry weight (CDW). The CDW was determined after lyophilization of cells obtained by centrifuging 10 ml of culture broth at 8,000 g for 15 min. The lyophilized cell pellet was analyzed for the fatty acid concentrations. The supernatants of the culture broth were analysed for residual sugars and (NH_4_)_2_SO_4_. The residual sugar concentrations were measured by high-performance liquid chromatography (HPLC) as previously described [23]. Residual ammonia concentrations were determined by the Ammonia Assay kit (Sigma-Aldrich) according to the manufacturer’s instructions. For the analysis of total lipids, fatty acids were converted to methyl esters (FAMEs) by methanolysis followed by gas chromatography (GC) as previously described [23]. The fatty acids were identified and quantified by comparison to standard FAMEs. Fatty acid content was defined as the percentage of the ratio of fatty acids to cell dry weight (% CDW).

### Saccharification of unbleached kraft hardwood pulp

Enzymatic saccharification was performed in 2 liter flasks at 45°C on a rotary shaker at 200 rpm. Lignocellulose-degrading enzymes loaded were Pentopan, Viscozyme (23 mg protein mL^-1^, determined by Bio-Rad protein assay), Celluclast (48 mg protein mL^-1^) and Novozyme 188 (57 mg protein mL^-1^). At given duration times, the slurry was withdrawn, centrifuged at 8,000 g for 20 min and the supernatant was used for subsequent analysis and fermentation.

### Phenotypic testing of carbon source utilization

Carbon assimilation profiles of the strains were evaluated using Phenotype MicroArray PM1 & 2 carbon panels (Biolog) by following the manufacturer’s instructions [38]. The reduction of the redox dye tetrazolium during respiration due to carbon catabolism causes the formation of a purple color in the well. Following incubation, the utilization of the sole substrates was measured spectrophotometrically at 595 nm, using a FLUOstar OPTIMA plate reader (BMG Labtech).

### Accession codes

The nucleotide sequences of pXsp8, pX0, pX1, pX2, pX3 and pX4 reported in this study were deposited in the GenBank database under accession numbers KC817027, KC817028, KC817029, KC817030, KC817031 and KC817032, respectively.

## Abbreviations

TAG: Triacylglycerol; CDW: Cell dry weight; OD: Optical density; C/N: Carbon to nitrogen ratio; ORF: Open reading frame; CBD: Cellulose-binding domain; xylA: Xylose isomerase; xylB: Xylulokinase; wt: Wild type; PCR: Polymerase chain reaction; HPLC: High performance liquid chromatography; GC: Gas chromatography; FAME: Fatty acid methyl ester.

## Competing interests

The authors declare no competing financial interests. The strategy described in this paper has been included in a patent publication (International publication NO. WO 2010/147642 A1).

## Authors’ contributions

KK conducted all the experiments, carried out the strain construction, the flask cultivations, the enzymatic saccharification and the genome analysis, analysed the results and wrote the manuscript. SJW performed the fermentor experiments, analysed the results. AJS conceived the study, helped plan and oversaw manuscript drafting. All authors read and approved the final manuscript.

## Supplementary Material

Additional file 1: Table S1 Growth and TAG production as fatty acids of *R. opacus* transformants on xylose. **Table S2.** Strains and plasmids used in this study. **Table S3.** Primers used in this study. Click here for file

Additional file 2: Figure S1 Carbon utilization profiles of *R. opacus* strains. Turbidity data is a measure of bacterial growth, indicative of substrate utilization. The order of the carbon sources is the rank of the growth on 190 substrates for PD630. **Figure S2.** Comparison of ORF-1 in pXsp8 insert with several xylose isomerases at the deduced amino acid sequence level using the CLUSTALW program. Hyg-XI, xylose isomerase of *S. hygroscopicus* accession no. AEY87893; Ave-XI, xylose isomerase of *S. avermitilis* accession no. NP_828358; Cha-XI, xylose isomerase of *S. chartreusis* accession no. ZP_09957234; Coe-XI, xylose isomerase of *S. coelicolor* accession no. NP_625460; Sp8-O1, ORF-1 of the 3603-bp insert. **Figure S3.** Comparison of ORF-2 in pXs8 insert with several xylulose kinases and cellulose-binding proteins at the deduced amino acid sequence level using the CLUSTALW program. Cha-XK, xylulose kinase of *S. chartreusis* accession no. ZP_09957235; Coe-XK, xylulose kinase of *S. coelicolor* accession no. NP_733525; Ave-XK, xylulose kinase of *S. avermitilis* accession no. NP_828357; Hyg-XK, xylulose kinase of *S. hygroscopicus* accession no. AEY87894; Sp8-O2, ORF-2 of the 3603-bp insert; Hyg-CB, cellulose-binding protein of *S. hygroscopicus* accession no. AEY91743; Ave-CB, cellulose-binding protein of *S. avermitilis* accession no. NP_824035; Cha-CB, cellulose-binding protein of *S. chartreusis* accession no. ZP_09956458; Coe-CB, cellulose-binding protein of *S. coelicolor* accession no. NP_629535. **Figure S4.** Construction of plasmid pX0, pX1, pX2, pX3 and pX4. These plasmids contain the NG2 origin of replication from pEP2, the RP4 *mob* element from pSUP301, a spectinomycin resistance marker derived from the omega interposon and the *trc* promoter from pTrc99A^47^. **Figure S5.** Elucidation of the molecular targets involved in improvement of xylose metabolism in *R. opacus* PD630. (a,b) Growth of *Rhodococcus* isolates on LB plates. (c,d) Detection of the plasmid DNA in *R. opacus* derivatives. Click here for file
