# Supplementary material for: Engineering xylose metabolism in triacylglycerol-producing Rhodococcus opacus for lignocellulosic fuel production
Source: Biotechnol Biofuels. 2013 Sep 16;6:134. doi: 10.1186/1754-6834-6-134 (PMC3848844; doi:10.1186/1754-6834-6-134)
Supplement: Additional file 1: Table S1 — Growth and TAG production as fatty acids of R. opacus transformants on xylose. Table S2. Strains and plasmids used in this study. Table S3. Primers used in this study. [file 1754-6834-6-134-S1.pdf]

Table S1 Growth and TAG production as fatty acids of *R. opacus* transformants on xylose

Strain	Growth		Fatty acid production	
	OD_{660}	CDW, g l^{-1}	% CDW	g l^{-1} of culture
Xsp1	10.0 ± 1.8	3.7 ± 0.9	32.7 ± 0.6	1.2 ± 0.3
Xsp8	13.6 ± 1.1	5.3 ± 0.5	39.0 ± 7.8	2.1 ± 0.5
Xsp10	10.5 ± 0.8	4.3 ± 0.2	30.7 ± 3.1	1.2 ± 0.2
Xsp12	12.7 ± 1.1	4.9 ± 0.5	32.0 ± 3.6	1.6 ± 0.3
Xsp23	10.2 ± 1.6	5.1 ± 1.2	9.3 ± 1.5	0.5 ± 0.1
Xsp26	11.3 ± 1.2	5.0 ± 0.5	8.7 ± 1.5	0.4 ± 0.1
Xsp33	12.8 ± 4.9	6.5 ± 4.5	10.0 ± 2.0	0.6 ± 0.3
Xsp37	12.0 ± 3.1	6.3 ± 1.4	10.3 ± 1.5	0.7 ± 0.1

The strains were grown in modified defined medium containing 40 g l^{-1} xylose and 1.4 g l^{-1} $(NH_4)_2SO_4$ supplemented with gentamicin in flask cultures for 6 days. Initial inoculum densities were adjusted to obtain an OD_{660} of 0.3. Data represent the results of triplicate experiments, ±s.d.

Table S2 Strains and plasmids used in this study

Strain or plasmid	Description	Reference
Strains		
Rhodococcus opacus		
PD630	Wild type	[20]
Xsp1	Gm^R; obtained by transformation of pAL358 carrying DNA fragments from *S. padanus* into PD630	This study
Xsp8	Gm^R; obtained by transformation of pAL358 carrying DNA fragments from *S. padanus* into PD630	This study
Xsp10	Gm^R; obtained by transformation of pAL358 carrying DNA fragments from *S. padanus* into PD630	This study
Xsp12	Gm^R; obtained by transformation of pAL358 carrying DNA fragments from *S. padanus* into PD630	This study
Xsp23	Gm^R; obtained by transformation of pAL358 carrying DNA fragments from *S. padanus* into PD630	This study
Xsp26	Gm^R; obtained by transformation of pAL358 carrying DNA fragments from *S. padanus* into PD630	This study
Xsp33	Gm^R; obtained by transformation of pAL358 carrying DNA fragments from *S. padanus* into PD630	This study
Xsp37	Gm^R; obtained by transformation of pAL358 carrying DNA fragments from *S. padanus* into PD630	This study
Xsp8C	Spontaneous plasmid (pXsp8)-cured strain of Xsp8	This study
Xsp8X0-1 to -6	Spec^R; obtained by transformation of pX0 into Xsp8C	This study
Xsp8X1-1 to -6	Spec^R; obtained by transformation of pX1 into Xsp8C	This study
Xsp8X2-1 to -6	Spec^R; obtained by transformation of pX2 into Xsp8C	This study
Xsp8X3-1 to -6	Spec^R; obtained by transformation of pX3 into Xsp8C	This study
Xsp8X4-1 to -6	Spec^R; obtained by transformation of pX4 into Xsp8C	This study
PD630X0-1 to -6	Spec^R; obtained by transformation of pX0 into PD630	This study
PD630X1-1 to -6	Spec^R; obtained by transformation of pX1 into PD630	This study
PD630X2-1 to -6	Spec^R; obtained by transformation of pX2 into PD630	This study
PD630X3-1 to -6	Spec^R; obtained by transformation of pX3 into PD630	This study
PD630X4-1 to -6	Spec^R; obtained by transformation of pX4 into PD630	This study
Streptomyces padanus		
MITKK-103	Wild type	[28]
Escherichia coli		
TOP10	F$^-$ *mcrA* Δ*(mrr-hsdRMS-mcrBC)* φ*80lacZ*Δ*M15* Δ*lacX74 recA1 araD139* Δ*(ara-leu)7697 galU galK rpsL (*Str^R*) endA1 nupG*	Invitrogen
Plasmids		
pCR-Blunt II-TOPO	TOPO cloning vector for bacterial expression, Km^R	Invitrogen
pAL358	*Rhodococcus*/*E. coli* shuttle vector, Gm^R	[48]
pXsp8	pAL358 derivative with 3603-bp *Bam*HI insert containing *xylA* and *xylB* from *S. padanus*	This study
pAL307	*Rhodococcus*/*E. coli* shuttle vector, Spc^R	[47]
pX0	pAL307 derivative carrying 125-bp *Bam*HI-*Spe*I insert without *xylA* and/or *xylB* from pXsp8	This study
pX1	pAL307 derivative with 1475-bp *Bam*HI-*Spe*I insert containing *xylA* from pXsp8	This study
pX2	pAL307 derivative with 2064-bp *Bam*HI-*Spe*I insert containing *xylB* from pXsp8	This study
pX3	pAL307 derivative with 3537-bp *Bam*HI-*Spe*I insert containing *xylA* and *xylB* from pXsp8	This study
pX4	pAL307 derivative with 2826-bp *Bam*HI-*Spe*I insert containing *xylA* and *xylB* without cellulose-binding domain from pXsp8	This study

Str^R, streptomycin resistance; Gm^R, gentamicin resistance; Spec^R, spectinomycin resistance; Km^R, kanamycin resistance.

Table S3 Primers used in this study

Primer	Sequence (5'-3')	Purpose
3603-*xylA*-U	GGATCCGTGGAAGACATCTGTCGA	Construction of pX0, pX1, pX3 and pX4
3603-*xylA*-D1	GGACTAGTGGAGCATGGCGAGGTGACGCA	Construction of pX1
3603-*xylA*-D2	CTGCAGGAGCATGGCGAGGTGACGCA	Construction of pX3 and pX4
3603-*xylB*-U1	GGATCCGGTGTCTTCGACCACAAGGT	Construction of pX2
3603-*xylB*-D	GGACTAGTGGAACCCTATCGGGTCTGCT	Construction of pX2 and pX3
3603-*xylB*-U2	CTGCAGTTTCAGCTGGCACGACAGGT	Construction of pX3 and pX4
3603-*xylB#*-D	GGACTAGT**CTA**CTGGACCGGCCGTCCGGACAGCCGT	Construction of pX4
3603-*dele*-D	GGACTAGTGGCTCCTTGTTCCGTACGA	Construction of pX0
pAL358-M13R0	GCACCTGTCCTACGAGTTGCA	Sequencing of pXsp8
pAL358-M13F0	CCTCTAGATGCATGCTCGA	Sequencing of pXsp8
pXsp8-F2	GCATCGACATCGAAGTCCTCGA	Sequencing of pXsp8
pXsp8-F3	GGTCGATGTGGAAGAGCT	Sequencing of pXsp8
pXsp8-F4	CGAAGGTGAACCTGTCCT	Sequencing of pXsp8
pXsp8-F5	CCTTTCTCCAAGGGCACGT	Sequencing of pXsp8
pXsp8-F6	CCAGCAGGTGCGTAACGA	Sequencing of pXsp8
pXsp8-F7	GCTTTGTCCCGTTCTAGCGT	Sequencing of pXsp8
pAL358-1164f	GCAACTGGTCCAGAACCT	Sequencing of pXsp8
pAL358-1754f	CGTGCAAGCAGATTACGGT	Sequencing of pXsp8
pAL358-2351f	CCACCAGCTATCCTTCTTGCA	Sequencing of pXsp8
pAL358-3037f	CCTGGTCGTAGACGTTGACCA	Sequencing of pXsp8
pAL358-3602f	CGTGTGCTCTGGATTGTCCA	Sequencing of pXsp8
pAL358-4320f	CCTCTGTTCTCCTAGACCT	Sequencing of pXsp8
pAL307-411f	GCTTGGCCAGGTTCGTCTCGA	Sequencing of pX2 and pX3
pAL307-638r	CCCAGCAGGTGCGTAACGA	Sequencing of pX3
pAL307-1062f	GCGTGCAGTTCAGGGTGCA	Sequencing of pX2, pX3 and pX4
pAL307-1649f	CGGACATCGTTCCAGAGCA	Sequencing of pX2, pX3 and pX4
pAL307-2099f	GGTCATGACGTCAGCATAGGA	Sequencing of pX3 and pX4
pAL307-2680f	CCGTTGAGGTCGATGTGGA	Sequencing of pX1, pX3 and pX4
pAL307-2953r	GCATGAAGGAGGCGTTCGACCT	Sequencing of pX1, pX3 and pX4
pAL307-3137f	GGGTGAAGAGGTTCGTGGT	Sequencing of pX1, pX3 and pX4
pAL307-3745f	GCACAGCCATACCACAGCT	Sequencing of pX0, pX1, pX2, pX3 and pX4
pAL307-4376f	GCTGGCTCGAAGATACCTGCA	Sequencing of pX0, pX1, pX2, pX3 and pX4
pAL307-4897f	GCGTCACCTTCATGGTGGT	Sequencing of pX0, pX1, pX2, pX3 and pX4
pAL307-5572f	GGGTTCGTGTAGACTTTCCT	Sequencing of pX0, pX1, pX2, pX3 and pX4
pAL307-6359f	CCACGGTTCAGTCACACGA	Sequencing of pX0, pX1, pX2, pX3 and pX4
pAL307-7073f	GCTCTGGATTGTCCAAGGA	Sequencing of pX0, pX1, pX2, pX3 and pX4
pAL307-7672f	GGGTCTCATACCGTAAGCA	Sequencing of pX0, pX1, pX2, pX3 and pX4

The restriction sites introduced into primers are underlined, and the stop codon is highlighted in bold.
