# Supplementary material for: Engineering xylose metabolism in triacylglycerol-producing Rhodococcus opacus for lignocellulosic fuel production
Source: Biotechnol Biofuels. 2013 Sep 16;6:134. doi: 10.1186/1754-6834-6-134 (PMC3848844; doi:10.1186/1754-6834-6-134)
Supplement: Additional file 2: Figure S1 — Carbon utilization profiles of R. opacus strains. Turbidity data is a measure of bacterial growth, indicative of substrate utilization. The order of the carbon sources is the rank of the growth on 190 substrates for PD630. Figure S2. Comparison of ORF-1 in pXsp8 insert with several xylose isomerases at the deduced amino acid sequence level using the CLUSTALW program. Hyg-XI, xylose isomerase of S. hygroscopicus accession no. AEY87893; Ave-XI, xylose isomerase of S. avermitilis accession no. NP_828358; Cha-XI, xylose isomerase of S. chartreusis accession no. ZP_09957234; Coe-XI, xylose isomerase of S. coelicolor accession no. NP_625460; Sp8-O1, ORF-1 of the 3603-bp insert. Figure S3. Comparison of ORF-2 in pXs8 insert with several xylulose kinases and cellulose-binding proteins at the deduced amino acid sequence level using the CLUSTALW program. Cha-XK, xylulose kinase of S. chartreusis accession no. ZP_09957235; Coe-XK, xylulose kinase of S. coelicolor accession no. NP_733525; Ave-XK, xylulose kinase of S. avermitilis accession no. NP_828357; Hyg-XK, xylulose kinase of S. hygroscopicus accession no. AEY87894; Sp8-O2, ORF-2 of the 3603-bp insert; Hyg-CB, cellulose-binding protein of S. hygroscopicus accession no. AEY91743; Ave-CB, cellulose-binding protein of S. avermitilis accession no. NP_824035; Cha-CB, cellulose-binding protein of S. chartreusis accession no. ZP_09956458; Coe-CB, cellulose-binding protein of S. coelicolor accession no. NP_629535. Figure S4. Construction of plasmid pX0, pX1, pX2, pX3 and pX4. These plasmids contain the NG2 origin of replication from pEP2, the RP4 mob element from pSUP301, a spectinomycin resistance marker derived from the omega interposon and the trc promoter from pTrc99A47. Figure S5. Elucidation of the molecular targets involved in improvement of xylose metabolism in R. opacus PD630. (a,b) Growth of Rhodococcus isolates on LB plates. (c,d) Detection of the plasmid DNA in R. opacus derivatives. [file 1754-6834-6-134-S2.pdf]

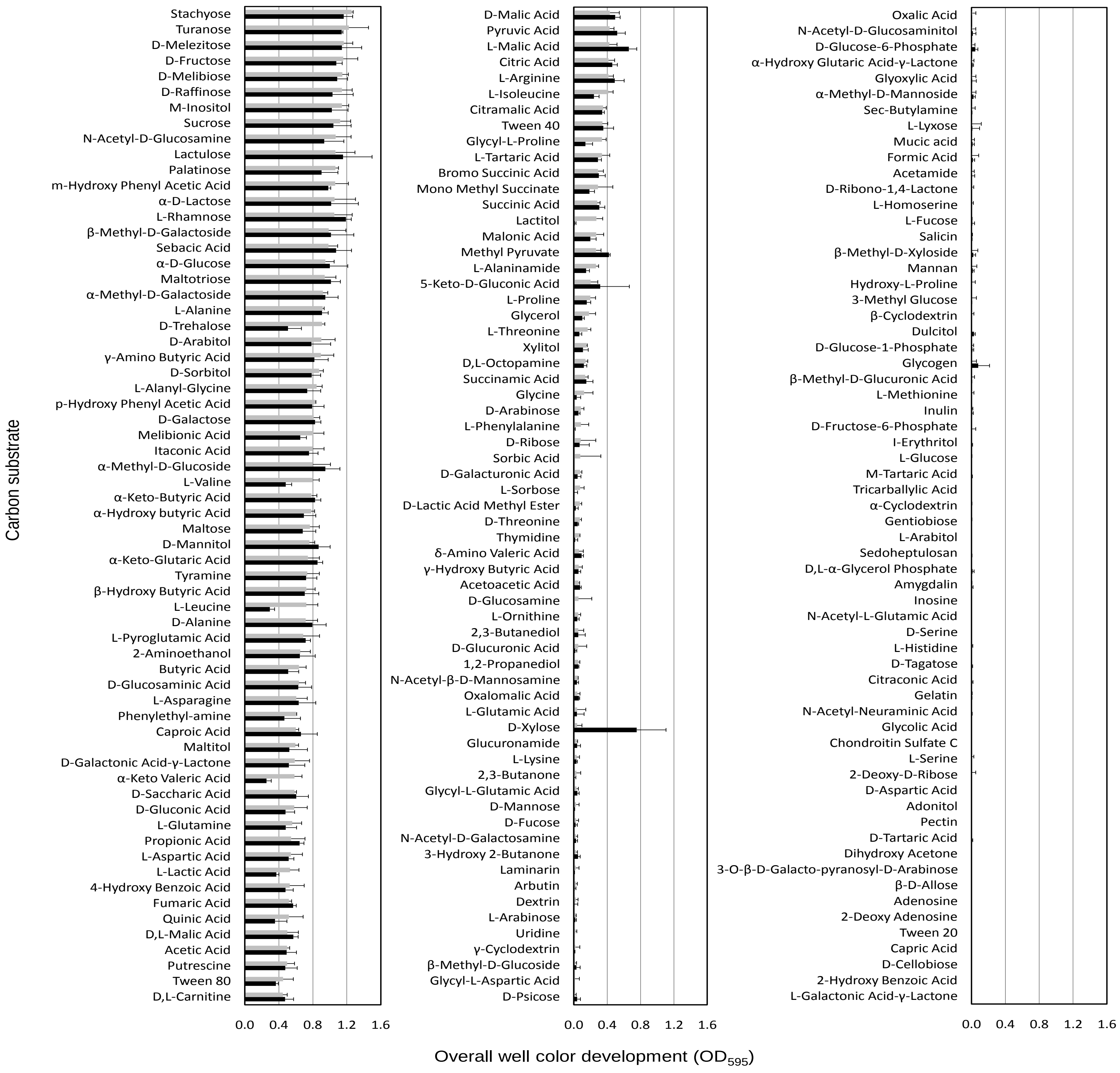

Figure S1 Carbon utilization profiles of *R. opacus* strains. Turbidity data measure bacterial growth, indicative of substrate utilization. Washed cells grown on LB plates for 3 days were suspended in 28 ml IF-0a GN/GP Base Inoculating Fluid (Biolog) and 0.3 ml Redox Dye Mix D (Biolog) and adjusted to an OD_{660} of 0.01. Aliquots of 50 µl the mixture were dispensed into each well of the BIOLOG PM microplates. Data are based on the absorbance following incubation of the plates for 5 days. The well color development for each sample was calculated as the mean value of the OD_{595} in the three replicates of each substrate, subtracted of the average OD_{595} in the background wells. The order of the carbon sources is the rank of the growth on 190 substrates for PD630. Error bars show the standard deviations.

```
Hyg-XI   MNYQPTPEDRFTFGLWTVGWQGRDPFGDATRRALDPVETVQRLAELGAHGVTFHDDDLIPFGSSDTERESHIKRFRQALD
Ave-XI   MNYQPTPEDRFTFGLWTVGWQGRDPFGDATRRALDPVETVQRLAGLGAHGVTFHDDDLIPFGSSDTERESHIKRFRQALD
Cha-XI   MSYQPTPEDRFTFGLWTVGWQGRDPFGDATRRALDPVETVQRLAELGAHGVTFHDDDLIPFGSSDTEREAHIKRFRQALD
Coe-XI   MNYQPTPEDRFTFGLWTVGWQGRDPFGDATRQALDPAESVRRLSELGAYGVTFHDDDLIPFGSSDTERESHIKRFRQALD
Sp8-O1   MSFQPTPEDRFTFGLWTVGWQGRDPFGDATRPALDPVETVQRLAELGAYGVTFHDDDLIPFGSSDTERESHIKRFRQALD  80
         *.:**************************** ****.*:*:**: ***:********************:**********

Hyg-XI   ATGMTVPMATTNLFTHPVFKDGAFTANDRDVRRYALRKTIRNIDLAAELGAKTYVAWGGREGAESGAAKDVRSALDRMKE
Ave-XI   ATGMAVPMATTNLFTHPVFKDGAFTANDRDVRRYALRKTIRNIDLAAELGAKTYVAWGGREGAESGAAKDVRVALDRMKE
Cha-XI   ATGMKVPMATTNLFAHPVFKDGAFTANDRDVRRYALRKTIRNIDLAVELGAQVYVAWGGREGAESGAAKDVRAALDRMKE
Coe-XI   ATGMKVPMATTNLFTHPVFKDGAFTANDRDVRRYALRKTIRNIDLAVELGASVYVAWGGREGAESGAAKDVRDALDRMKE
Sp8-O1   ATGMTVPMATTNLFTHPVFKDGGFTANDRDVRRYALRKTIRNIDLAAELGAKTYVAWGGREGAESGGAKDVRDALDRMKE 160
         **** *********:*******.***********************.****..*************.***** *******

Hyg-XI   AFDLLGEYVTSQGYDLRFAIEPKPNEPRGDILLPTVGHALAFIERLERPELYGVNPEVGHEQMAGLNFPHGIAQALWAGK
Ave-XI   AFDLLGEYVTAQGYDLRFAIEPKPNEPRGDILLPTVGHALAFIERLERPELYGVNPEVGHEQMAGLNFPHGIAQALWAGK
Cha-XI   AFDLLGEYVTSQGYDLRFAIEPKPNEPRGDILLPTVGHALAFIERLERPELYGVNPEVGHEQMAGLNFPHGIAQALWAGK
Coe-XI   AFDLLGEYVTEQGYDLKFAIEPKPNEPRGDILLPTVGHALAFIERLERPELYGVNPEVGHEQMAGLNFPHGIAQALWAGK
Sp8-O1   AFDLLGEYVIAQGYDLRFAIEPKPNEPRGDILLPTVGHALAFIERLERPELYGVNPEVGHEQMAGLNFPHGIAQALWAGK 240
         *********  *****:***************************************************************

Hyg-XI   LFHIDLNGQSGIKYDQDLRFGAGDLRSAFWLVDLLESAGYEGPRHFDFKPPRTEDLDGVWASAAGCMRNYLILKERAAAF
Ave-XI   LFHIDLNGQSGIKYDQDLRFGAGDLRAAFWLVDLLESAGYEGPKHFDFKPPRTEDLDGVWASAAGCMRNYLILKERTAAF
Cha-XI   LFHIDLNGQSGIKYDQDLRFGAGDLRAAFWLVDLLESAGYTGPRHFDFKPPRTEDLDGVWASAAGCMRNYLILKERAAAF
Coe-XI   LFHIDLNGQSGIKYDQDLRFGAGDLRAAFWLVDLLERAGYAGPRHFDFKPPRTEDFDGVWASAAGCMRNYLILKDRAAAF
Sp8-O1   LFHIDLNGQSGIKYDQDLRFGAGDLRAAFWLVDLLETAGYEGPRHFDFKPPRTEDFDGVWASAAGCMRNYLILKDRAAAF 320
         **************************:********* *** **:***********:******************:*:***

Hyg-XI   RADPEVQEALRASRLDQLAQPTAADGLEDLLADRAAFEDFDVEAAAARGMAFERLDQLAMDHLLGARG
Ave-XI   RADPEVQEALRAARLDELAQPTAGDGLTALLADRTAFEDFDVEAAAARGMAFEQLDQLAMDHLLGARG
Cha-XI   RADPEVQEALRAARLDELAQPTAADGLQALLADRSAFESFDVEAAAARGMAFEHLDQLAMDHLLGARG
Coe-XI   RADPQVQEALAAARLDELARPTAEDGLAALLADRSAYDTFDVDAAAARGMAFEHLDQLAMDHLLGAR-
Sp8-O1   RADPEVQEALRAARLDQLAQATAADGLDALLADRSAFEDFDVDAAAARGMAFEHLDQLAMDHLLGARG 388
         ****:***** *:***:**:.** ***  *****:*:: ***:**********:*************
```

Figure S2 Comparison of ORF-1 in pXsp8 insert with several xylose isomerases at the deduced amino acid sequence level using the CLUSTALW program. Hyg-XI, xylose isomerase of *S. hygroscopicus* accession no. AEY87893; Ave-XI, xylose isomerase of *S. avermitilis* accession no. NP_828358; Cha-XI, xylose isomerase of *S. chartreusis* accession no. ZP_09957234; Coe-XI, xylose isomerase of *S. coelicolor* accession no. NP_625460; Sp8-O1, ORF-1 of the 3603-bp insert. Numbering of the amino acids starts at the N-terminus of all proteins. The introduction of gaps is indicated by dashes. Asterisks, colons and periods indicate identical, strongly similar and weakly similar conserved residues, respectively, among all of these enzymes.

```
         *************:***********.:**:***********::* ************* *** ***:**:****:*:******
Cha-XK   MSAAEGPLVVGVDSSTQSTKALVVDVSTGRVVASGQAPHTVSSGSGRESDPRQWWDALCEALRQCGDAAHEAAAVSVGGQQHG
Coe-XK   MSAAEGPLVVGVDTSTQSTKALVVDAATGRVVASGQAPHTVSSGTGRESDPRQWWDALGEALSQCGEAAREAAAVSVGGQQHG
Ave-XK   MSAAEGPLVVGVDTSTQSTKALVVDASTGRVVASGQAPHTVSAGAGRESDPRQWWDALCEALHQCGDAAHEAAAISVGGQQHG
Hyg-XK   MSAAEGPLVVGVDTSTQSTKALVVDAATGQVVASGQAPHTVSSGAGRESDPRQWWDALCEALRQCGDAAHEAAAVSIGGQQHG
Sp8-O2   MSAAEGPLVVGVDTSTQSTKALVVDAATGQVVASGQAPHTVTTGGGRESDPRQWWDALCEALRQCGDAAHEAAAISVGGQQHG  83

         ***** :*:***************** ** :****.* *********..***.:*****:******.**. ************
Cha-XK   LVTLDDRGEPVRPALLWNDVRSAPQARRLTEELGGAKFWAERTGSVPAASFTVTKWAWLAEHEPEAARATKAVRLPHDYLTER
Coe-XK   LVTLDARGEPVRPALLWNDVRSAPQARRLIDELGGAKAWAERTGSVPSASFTVTKWAWLTEHEPEAARAVKAVRLPHDYLTER
Ave-XK   LVTLDAHGDPVRPALLWNDVRSAPQARRLIEELGGPKAWAERTGSVPGPSFTVTKWAWLTEHEPEAVRATAAVRLPHDYLTER
Hyg-XK   LVTLDERGEPVRPALLWNDVRSAPQAGRLVEELGGPKAWAERTGSVPGASFTVTKWAWLAEHEPEAVRATRAVRLPHDYLTER
Sp8-O2   LVTLDERGEPVRPALLWNDVRSAPQARRLVEELGGPKAWAERTGSVPGASFTASKWAWLAEHEPEAARATRAVRLPHDYLTER 166

         ***:..*****.********.** ***: *:::.**************:.*****.*.**********.*************:
Cha-XK   LTGQGTTDRGDASGTGWWASGTERYDEEILAQVGLDPALLPRVVRPGEIAGTVRDSHDLPFSKGTLVAPGTGDNAAAALGLGV
Coe-XK   LTGEGTTDRGDVSGTGWWASGTEAYDEEILARVALDPALLPRVVRPGEVAGTVRDGHGLPFSKGTLVAAGTGDNAAAALGLGL
Ave-XK   LTGQGTTDRGDVSGTGWWASATESYDEEVLTHVGLDPALLPRVVRPGEVVGTVRDSHDLPFSKGTLVAPGTGDNAAAALGLGL
Hyg-XK   LTGQGTTDRGDASGTGWWASGTEAYDEETLAHVGLDPALLPRVVRPGEVAGTVRDSHDLPFSKGTLVAPGTGDNAAAALGLGL
Sp8-O2   LTGQAVTDRGDASGTGWWASGTERYDEDTLAHLGLDPALLPRVVRPGEVAGTVRDGHGLPFSKGTLVASGTGDNAAAALGLGL 249

          **.**:*******.**** :**:**:*******************************:********* .*  *****:*****
Cha-XK   RPGVPVMSLGTSGTVYAVSQRRPADPSGTVAGFADARGDWLPLACTLNCTLAVDRVATLLGLDREAVEPGTAVTLLPYLDGER
Coe-XK   RPGVPVMSLGTSGTAYAVSQRRPADPTGTVAGFADARGDWLPLACTLNCTLAVDRVASLLGLDREAVEPGTDVTLLPFLDGER
Ave-XK   RPGTPVLSLGTSGTVYAVSTRRPADPTGTVAGFADARGDWLPLACTLNCTLAVDRVAALLGLDREAVEPGTGVTLLPYLDGER
Hyg-XK   RPGTPVLSLGTSGTVYAVSRRRPTDPTGTVAGFADARGDWLPLACTLNCTLAVDRVAALLGLDREAVESGGSVTLLPYLDGER
Sp8-O2   LPGTPVLSLGTSGTVYAVSRHRPADPTGTVAGFADARGDWLPLACTLNCTLAVDRVATLLGLDREAVAPGGSVTLLPFLDGER 332

         **:**::**:********* *************:********** *** .:*********** ***************: .
Cha-XK   TPNLPNASGLLHGLRHDTTAGQLLQAAYDGAVHSLLGALDLVLDEDADPSAPLLLIGGGARGTAWQQTVRRLSGRPVQVPEAK
Coe-XK   TPNLPHSSGLLHGLRHDTTAGQLLQAAYDGAVHSLLGALDLVLDADADPSAPLLLIGGGARGTAWQQTVRRLSGRPVQIPEAR
Ave-XK   TPDLPHASGLLHGLRHDTTGGQLLQAAYDGAVHALLGALDLVLDADADRSAPLLLIGGGARGTAWQQTVRRLSGRPVQVPEAR
Hyg-XK   TPNLPHASGVLHGLRHDTTAGQLLQAAYDGAVHALLGALDLVLDEDADRDTPLLLIGGGARGTAWQQTVRRLSGRPVQVPEAR
Sp8-O2   TPDLPNASGLLHGLRHDTTPGQLLQAAYDGAVHSLLGALDLVLDEDADRDSPLLLIGGGARGHAWQQTVRRLSGRPVQILRLA 415
Hyg-CB     MSDTSPYGFELVRRGYDRAQVDERISKLVSDRDSALARITALEKRIEELHLETQNAQAQITDAEPSYAGLGARVEKILRLA  81
Ave-CB     MSDTSPYGFELVRRGYDRAQVDERISKLVSDRDSALARITALEKRIEELHLETQNAQAQVTDAEPSYAGLGARVEKILRLA  81
Cha-CB     MSDTSPYGFELVRRGYDRAQVDERISKLVSDRDSALARITALEKRIEELHLETQNAQAQISDAEPSYAGLGARVEKILRLA  81
Coe-CB     MSDTSPYGFELVRRGYDRAQVDERISKLVSDRDSALARITALEKRIEELHLETQNAQAQVNDAEPSYAGLGARVEKILRLA  81
               .:.  :. :*:.   .*: :   . . .   .    :.   *.  .:  *   .. *:    : :   *..*  :*****

         *  *     :*      .    * :   :.  ..   *.**.  :   . : ::..*    .:
Cha-XK   ELVALGAAAQAAGVLTGEDPAAVARRWNTAAGPVLDAVERDEETLARIAGVLSDAAPLLERPTDTH 481
Coe-XK   ELVALGAAAQAAGLLTGEDAAAVARRWNTAAGPVLDAVERDEATLNRITGVLSDAAPLLERDAASR 481
Ave-XK   ELVALGAAAQAAGVLTGEDPAAVARRWDTAHGPVLDAVERDEATLARISGVLSDAAPLLEREPDRR 481
Hyg-XK   ELVALGAAAQAAGLLTGEDPAAVARRWNTAAGPVLEAVERDRTTLDRITGVLSDAAPLLERGTDTE 481
Sp8-O2   EEEAKELREEARRAAEQHRELAESAAQQVRNDAESYATERKAKAEDEGSRIVEKAKGEASQLRADAQKDAQSKREEADALFEE 498
Hyg-CB   EEEAKELREEARRAAEQHRDLAESAAQQVRNDAESYAAERKAKAEDEGVRIVEKAKSDAAQLRAEAQKDAQSKREEADALFEE 164
Ave-CB   EEEAKDLREEARRASEQHRELAESAAQQVRNDAESFAADRKSKAEDEGVRIVEKAKSDASQLRQEAQKDAQSKREEADALFEE 164
Cha-CB   EEEAKDLREEARRAAEQHRELAESAAQQVRNDAESYSAERKAKAEDEGLRIVEKAKSDAAQLRAEAQKDAQSKREEADALFEE 164
Coe-CB   EEEAKDLREEARRAAEQHRELAESSAQQVRNDAESYAAERKAKAEDEGVRIVEKAKGDASQLRSEAQKDAQSKRDEADALFEE 164
         *****:*******:****:****:**********::::**:****** *******.:*:*** :*********:*******

Sp8-O2   TRAKAAQAAADFETNLAKRREQSERDLASRQQKAEKRLAEIEHRAEQLRLEAEKLRTDAERRARQTVETAQRQAEDIVADANA 581
Hyg-CB   TRAKAAQAAADFETNLAKRREQSERDLASRQQKAEKRLAEIEHRAEQLRLEAEKLRTDAERRARQTVETAQRQAEDIVADANA 247
Ave-CB   TRAKAAQAAADFETNLAKRREQSERDLASRQAKAEKRLAEIEHRAEQLRLEAEKLRTDAERRARQTVETAQRQAEDIVADANA 247
Cha-CB   TRAKAAQAAADFETNLAKRREQSERDLASRQAKAEKRLAEIEHRAEQLRLEAEKLRTDAERRARQTVETAQRQAEDIVADANA 247
Coe-CB   TRAKAAQAAADFETNLAKRREQSERDLASRQAKAEKRLAEIEHRAEQLRLEAEKLRTDAERRARQTVETAQRQSEDIVADANA 247
         ****************************** ****************************************:*********

Sp8-O2   KADRIRSESERELAALTNRRDSINAQLTNVREMLATLTGAAVAAAGT-TGEDEPVSRGVPAQQTR 645
Hyg-CB   KADRIRSESERELAALTNRRDSINAQLTNVREMLATLTGAAVAAAGT-STDDEPISRGVPAQQSR 311
Ave-CB   KADRIRSESERELAALTNRRDSINAQLTNVREMLATLTGAAVAAAGTPSTDDEPISRGVPAQQSR 312
Cha-CB   KADRIRSESERELAALTNRRDSINAQLTNVREMLATLTGAAVAAAGT-PAEDEPISRGVPAQQSR 311
Coe-CB   KADRIRSESERELAALTNRRDSINAQLTNVREMLASLTGAAVAAAPS--VEDESVSRGVPAQQSR 310
         ***********************************:********* :   :**.:********:*
```

Figure S3 Comparison of ORF-2 in pXs8 insert with several xylulose kinases and cellulose-binding proteins at the deduced amino acid sequence level using the CLUSTALW program. Cha-XK, xylulose kinase of *S. chartreusis* accession no. ZP_09957235; Coe-XK, xylulose kinase of *S. coelicolor* accession no. NP_733525; Ave-XK, xylulose kinase of *S. avermitilis* accession no. NP_828357; Hyg-XK, xylulose kinase of *S. hygroscopicus* accession no. AEY87894; Sp8-O2, ORF-2 of the 3603-bp insert; Hyg-CB, cellulose-binding protein of *S. hygroscopicus* accession no. AEY91743; Ave-CB, cellulose-binding protein of *S. avermitilis* accession no. NP_824035; Cha-CB, cellulose-binding protein of *S. chartreusis* accession no. ZP_09956458; Coe-CB, cellulose-binding protein of *S. coelicolor* accession no. NP_629535. Numbering of the amino acids starts at the N-terminus of all proteins. Sequences of xylulose kinases and cellulose-binding proteins are depicted in black and blue letters, respectively. The introduction of gaps is indicated by dashes. Asterisks, colons and periods indicate identical, strongly similar and weakly similar conserved residues, respectively, among all of these enzymes.

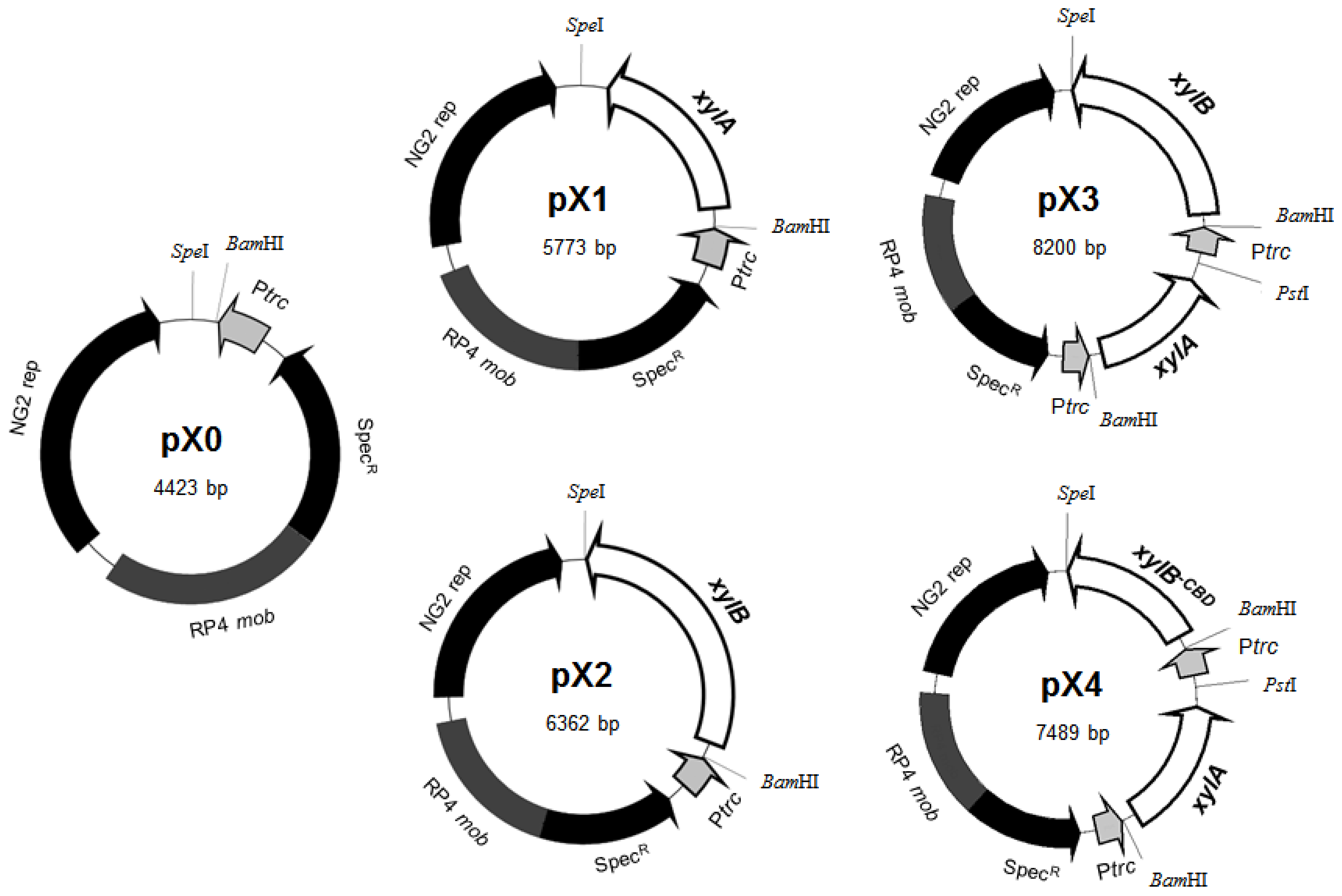

Figure S4 Construction of plasmid pX0, pX1, pX2, pX3 and pX4. These plasmids contain the NG2 origin of replication from pEP2, the RP4 *mob* element from pSUP301, a spectinomycin resistance marker derived from the omega interposon and the *trc* promoter from pTrc99A[47]. See for methods for details.

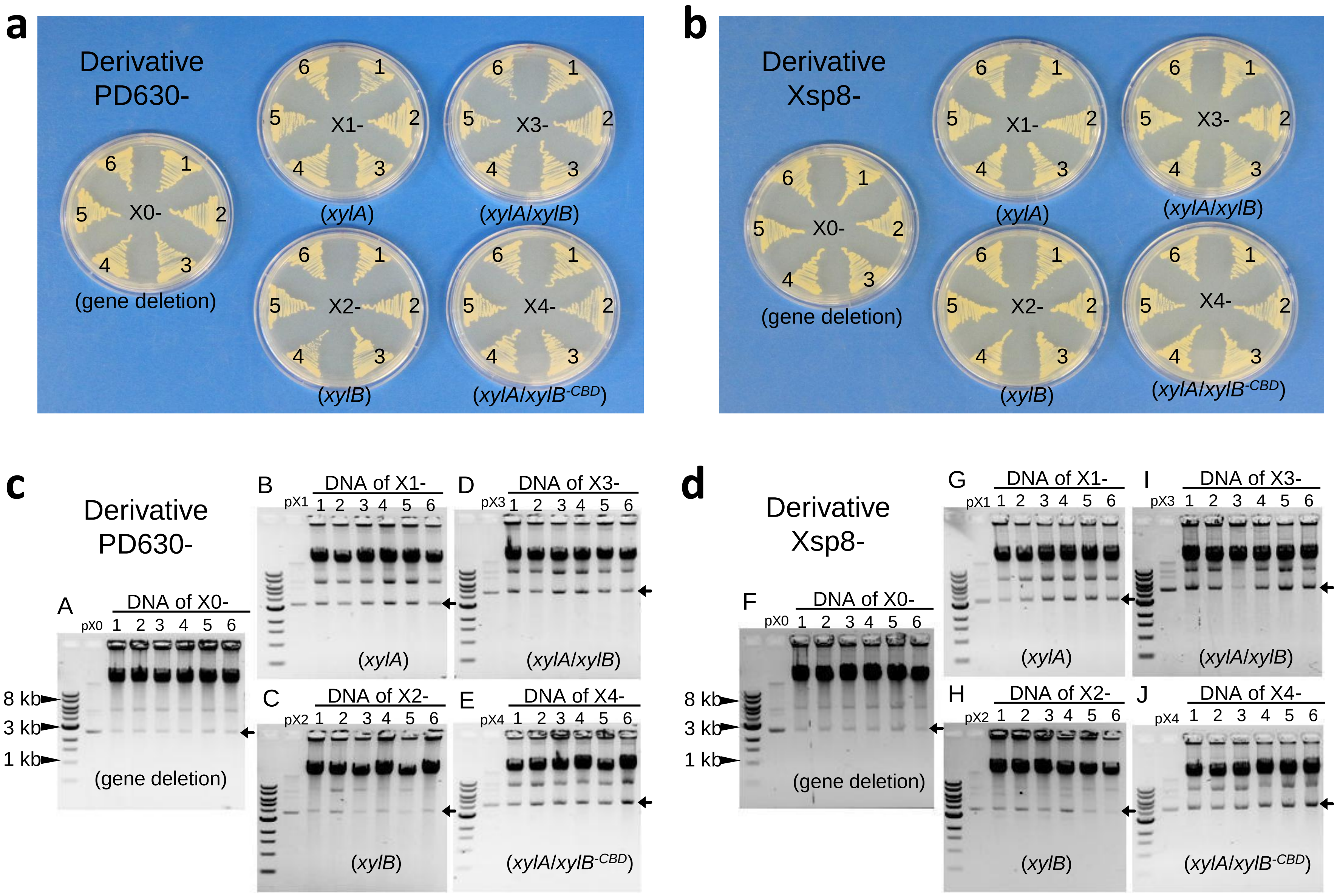

Figure S5 Elucidation of the molecular targets involved in improvement of xylose metabolism in *R. opacus* PD630. (**a,b**) Growth of *Rhodococcus* isolates on LB plates. Six colonies each plate in Figure 6a, b were randomly streaked onto LB plate and incubated for 3 days. The isolates were designated PD630X0-1 to -6, PD630X1-1 to -6, PD630X2-1 to -6, PD630X3-1 to -6 and PD630X4-1 to -6, respectively, from pX0, pX1, pX2, pX3 and pX4 plates in Figure 6a, and Xsp8X0-1 to -6, Xsp8X1-1 to -6, Xsp8X2-1 to -6, Xsp8X3-1 to -6 and Xsp8X4-1 to -6, respectively, from pX0, pX1, pX2, pX3 and pX4 plates in Figure 6b. (**c,d**) Detection of the plasmid DNA in *R. opacus* derivatives. Each strain was grown in LB medium supplemented with spectinomycin for 24 h in a flask. The total genomic DNA of each strain was prepared and subjected to 0.7% agarose gel electrophoresis. Arrows in panels A and F, pX0; arrows in panels B and G, pX1; arrows in panels C and H, pX2; arrows in panels D and I, pX3; arrows in panels E and J, pX4. Filled arrowheads indicate size of relevant markers (1 kb ladder).
